# Identifying sleep disturbances in Huntington’s disease using a simple disease-focused questionnaire

**DOI:** 10.1371/currents.RRN1189

**Published:** 2010-10-15

**Authors:** Anna O. G. Goodman, A. Jennifer Morton, Roger A Barker

**Affiliations:** ^*^Cambridge Centre for Brain Repair and ^†^University of Cambridge

## Abstract

Sleep disturbances have been shown to affect patients with various neurological diseases, including Huntington’s disease (HD). We therefore aimed to develop a sleep questionnaire that could be used by clinicians to help identify sleep disturbances in patients with the disease.

Design

A detailed questionnaire was used that was modelled on recent sleep questionnaires used for Parkinson’s disease patients, and developed after consultation with sleep specialists. This questionnaire contained 45 questions that focused on different sleep-related issues such as duration, quality of sleep, abnormal nocturnal behaviour and quality of life.

Setting

Questionnaires were either completed in the home environment or in clinic.

Participants

66 patients, 38 carers and 60 non-carers were recruited.

Measurements & Results

Various sleep-related difficulties were identified in a significantly greater proportion of HD patients compared to control subjects, with both quality and quantity of sleep being affected.

Conclusions

Disturbed sleep in HD may contribute towards the deterioration of the patient’s ability to do activities of daily living and have a significantly deleterious effect on the quality of life of both patients and carers. This simple questionnaire should aid the clinician by providing subjective insight into the patient’s sleep patterns that could enable more effective, individual-specific treatment to be instigated and ultimately improve quality of life.

INTRODUCTION 

      Sleep deficits in humans can cause serious neurological problems[1] and conversely, patients with neurodegenerative diseases such as Parkinson’s and Alzheimer’s disease often develop serious sleep disturbances[2] [3] [4] [5] although the nature of the abnormalities appears to vary between diseases.  Such disturbances can not only seriously affect the quality of life of the patient, carer and family[1] but can result in cognitive impairment, inattentiveness, poor memory, mood disorders and an increased risk of accidents[6] [7] and institutionalisation.  Therefore identifying abnormalities in sleep patterns could significantly impact on the optimal management of such patients.

      Huntington’s disease (HD) is an autosomal dominant neurodegenerative condition that affects approximately 1/20,000 individuals and runs a progressive course over 15-20 years, until death.  The main symptomatology is a triad of motor disturbances (primarily hyperkinesis), cognitive decline, and psychiatric symptoms, with the primary pathology in early disease being the selective degeneration of medium spiny neurons of the striatum[8] [9] However as the disease progresses, neurodegeneration becomes increasingly widespread and extends to other brain regions including the hypothalamus[9] [10], which may in turn lead to the expression of more poorly-characterised features of the disease such as weight loss and sleep disturbances[4] [11].

      In this last respect, polysomnography sleep studies have provided evidence of a progressively worsening sleep disorder in HD[12] [13], with awake electroencephalograms (EEGs) of HD patients showing a gradual slowing and diminution of amplitude as the disease progresses[14] [15] [16].  Disturbances in the stages of sleep in HD varies with disease stage[12] [16] [17] [18], but in routine clinical practice, is rarely taken into account in the measurement and assessment of patients with this condition.

      Therefore we have set about trying to assess the extent of the sleep problems in HD, using a simple questionnaire.  

MATERIALS & METHODS

      We classified the extent and variety of the sleep-related changes that occur in HD using a descriptive cross-sectional study of patients (covering all stages of the disease) attending our HD clinic. 

*Questionnaire design*

      A detailed questionnaire was used (Appendix 1) that was modelled on recent sleep questionnaires used for Parkinson’s disease patients, and developed after consultation with Dr. Gregory Stores (University of Oxford) and the Respiratory Support and Sleep Centre at Papworth Hospital, Cambridgeshire.  This questionnaire contained 45 questions that focused on different sleep-related issues such as duration, quality of sleep, abnormal nocturnal behaviour and quality of life.   

*Subjects*

      Over a six month period, patients at all stages of the disease attending the Brain Repair Centre (BRC) HD clinic were given the BRC HD Sleep Questionnaire to complete independently (*n = * 66).  Patients who were on sleep related medication were not included in the final analysis (*n = * 8).

      Two age and sex-matched control groups were used; (a) the first group was drawn from the immediate family of the patients, or their carer/s accompanying patients attending the clinic (*n = * 38); (b) a second group was made up from general members of the public who were associated with the University of Cambridge but were not associated with the patients, ie. ‘non-carers’ (*n = * 60).  All responding patients completed the questionnaire independently, without carer help.  

*Scoring system*

      To make this questionnaire more convenient for clinical use, a scoring system was required producing a total points score that would give the clinician an indication of the level of sleep disturbance in the assessed patient.  After the data had been analysed, a scoring system for the questionnaire was devised, allocating either 0, 1 or 2 points depending on the question. 

      Some questions were scored because they were significantly different in patients compared to control subjects were 1, 7, 8-11, 14, 15, 18, 19, 24, 27, 28 and 34.  These were allocated a score of 1 point.  Two questions that were not significantly different in this study were included in the scoring because previous studies^17^ had shown statistically significant differences in the parameter (Questions 4 and 6).  For Question 4, answering ‘≤3 hours’ or ‘≥11 hours’ scored 2 points, answering ‘4 hours’ and ‘10 hours’ scored 1 point.  In Question 5, answering ‘2+ hours’ scored 2 points, answering ‘1 hour’ scored 1 point.  In Question 6, answers of ‘6+ times’ scored 2 points, while answers of ‘4 times’ and ‘5 times’ scored 1 point.  A total of 16 scored questions were therefore used, with a maximum total score of 19 points (see Appendix 2).   

      Questions 12 (a-k and m-p), 13, 16, 20-23, 25, 26, 29 (a,c,e,h), 30-33, 35 and 39 were excluded from the questionnaire scoring system because they were not significantly different between groups.  Some components of Question 29 (b,d,f,g,i,j) were significantly different between patients and control subjects but were not used because they do not describe disturbed sleep and their response opposites, i.e. ‘restless’ ‘interrupted’, ‘dreamless’, ‘short’ and ‘light’ did not achieve significant results.  Further, although Questions 2, 12 (l), 17, 36-38, 40 and 41 were significantly different, they were not used.  They were omitted because with hindsight, either the question was considered to be ambiguous or, although the question provided additional informative details about the patient, it didn’t provide information relevant to sleep and/or disturbance in it.  

      The scoring system was validated by retrospectively calculating each respondent’s total HD sleep score.   

*Analysis*

      For analysis, control subjects were grouped as ‘carers’, ‘non-carers’ or as ‘combined’ control group which comprised of all the subjects from the carer and non-carer groups.  Since the carer control group was made up of partners and family members, it is likely that there is a higher frequency of disturbed sleep in this group than in the non-carer group.  For this reason, the non-carer control group was considered to be more representative of the general population and was therefore used for most statistical comparisons made during the analysis.

      Data were analysed using chi-square tests and the Fisher-exact test when the frequency of individuals answering questions fell below five.  A critical value for significance of *p <* 0.05 was used for all studies.  Data were expressed as mean ± standard deviation unless stated otherwise. 

RESULTS 

      The demographic data of patients and control subjects were not significantly different between any of the groups, Table 1.   

Table 1.  Demographic data for subjects used in the sleep disturbance questionnaire study.  

**Table:** 

	Sex	Mean age	TFC	UHDRS	MMSE	BDI
Group	M/F	years				
*Controls *- non-carers	24/36	51 (35-71)	-	-	-	-
carers	23/15	61 (33-80)	-	-	-	-
*Patients* - all	32/34	52.7 (27-74)	8.3 (2-13)	27.2 (0-65)	25.9 (16-30)	6.6 (0 – 22)
with TFC (>8)	19/13	51.6 (27–69)	11.6 (9-13)	15.2 (0-35)	27.5 (23-30)	6.2 (0-22)
(5-8)	8/12	52.6 (30-74)	6.9 (5-8)	33.2 (6-55)	25.2 (16-30)	7.2 (0-19)
(<5)	5/9	55.4 (39-73)	2.9 (2-4)	46.1 (22-65)	23.1 (19-27)	6.7 (0-19)
						

 Abbreviations: BDI, Beck Depression Inventory; F, female; M, male; MMSE, mini mental state examination; TFC, total functional capacity; UHDRS, Unified Huntington's Disease Rating Scale; “-” information not available.  All data are mean (range).  

Respondents, both patients and control subjects, did not always answer all of the questions.  Significant differences were found between patients and carers (in 12 questions), non-carer control groups (in 24 questions), and the combined control group (in 23 questions) (see Table 2).   

 Table 2. Significant results from sleep questionnaire showing responses in patients (n = 66), carer controls (n = 38), non-carer controls (n = 60) and combined controls (n = 98). p-values are given in brackets. * p<0.05, **p<0.01, ***p<0.001.

**Table:** 

Sleep Questionnaire - Significant Responses				
	Patients	Carers	Non-Carers	Combined
	*“Yes” number (%) of responses (p-value <)*			
2. What time do you normally wake up?	07.50	07.10 (0.01)**	07.06 (0.001)***	07.07 (0.001)***
5. Time taken to fall asleep?				
* One hour or more*	23.5	5.3 (0.02) *	8.3 (0.03)*	7.1 (0.01)**
7. Sleep changed over 5 years?				
*I need more sleep*	35.3	10.5 (0.01)**	20 (0.05)*	16.3 (0.01)**
8. Have difficulty falling asleep at night?	35.3	18.4 (0.07)	11.7 (0.01)**	14.3 (0.01)**
9. Have difficulty staying asleep at night?	33.8	18.4 (0.09)	13.3 (0.01)**	15.3 (0.01)**
10. Wake up for long periods during the night?	29.4	15.8 (0.12)	15.0 (0.05)*	15.3 (0.03)*
11. Wake up early in the morning, unable to return to sleep?	35.3	36.8 (0.87)	16.7 (0.02)*	24.5 (0.13)
14. Repeated jerking/twitching of arms or legs during sleep?	33.8	2.6 (0.001)***	10 (0.001)***	7.1 (0.001)***
15. Painful muscle cramps in arms/legs that wake you up?	25	18.4 (0.44)	5 (0.01)**	10.2 (0.01)**
17. Wander about at night?	11.8	7.9 (0.74)	1.7 (0.04)*	4.1 (0.07)
18. Get very agitated at night?	17.6	2.6 (0.03)*	5 (0.03)*	4.1 (0.01)**
19. Get up a lot to go to the toilet at night?	33.8	31.6 (0.81)	16.7 (0.03)*	22.4 (0.11)
24. Fidget a lot in bed?	60.3	26.3 (0.001)***	15 (0.001)***	19.4 (0.001)***
27. ‘Act out’ dreams eg) punch or shout while asleep?	11.8	0 (0.05)*	5 (0.22)	3.1 (0.03)*
28. Injure yourself or others while dreaming?	7.4	0 (0.16)	0 (0.06)	0 (0.01)**
29. b)How would you describe your sleep: *Deep*	38.2	55.3 (0.09)	63.3 (0.01)**	59.2 (0.01)**
d) *Long*	29.4	63.2 (0.001)***	55 (0.01)**	58.2 (0.001)***
f) M*any dreams*	19.1	34.2 (0.08)	43.3 (0.01)**	39.8 (0.01)**
g) *Uninterrupted*	26.5	42.0 (0.10)	46. (0.02)*	44. (0.02)*
i) *Still*	17.6	52.6 (0.001)***	60 (0.001) ***	57.1 (0.001)***
34. Find you are awake at night but asleep during the day?	16.8	2.6 (0.05)*	1.7 (0.01) **	2.0 (0.01) **
36. Take treatment for a medical condition?	47.1	28.9 (0.07)	25 (0.01) **	26.5 (0.01) **
37. Take treatment for a psychiatric problem?	13.2	0 (0.03) *	0 (0.01) **	0 (0.001)***
38. Smoke tobacco?	22.1	7.9 (0.10)	1.7 (0.001)***	4.1 (0.001)***
40. Go outdoors everyday or most days?	64.7	92.1 (0.01)**	91.7 (0.001)***	91.8 (0.001)***
41. Are you being treated for a sleep problem?	11.8	2.6 (0.15)	1.7 (0.04)*	2.0 (0.02)*

 Questions were grouped for convenience into themed subcategories.  These were quality of sleep (Questions 4-12, 29, 34) motor activity (Questions 14, 15, 17, 24) abnormal nocturnal behaviour (Questions 27, 28) and other aspects of disturbed sleep (Questions 13, 16, 18, 19, 21-23, 25, 26, 30-33, 35-41). 

*Quality of Sleep*

      A significantly greater proportion of HD patients compared to control subjects reported; difficulty in (i) falling asleep (χ^2^ (1) = 9.7, *p <* 0.01); (ii) maintaining sleep (χ^2^ (1) = 7.3, *p <* 0.01); (iii) taking an hour or more to get to sleep (χ^2^ (1) = 5.4, *p <* 0.05); (iv) needing ‘more sleep’ over the past 5 years (χ^2^ (1) = 3.7, * p <* 0.05); (v) being awake for long periods during the night (χ^2^ (1) = 3.8, *p <* 0.05); (vi) ‘often’ being awake at night but asleep during the day (χ^2^ (1) = 7.9, * p <* 0.01) and (vii) waking up early in the morning, unable to go back to sleep (χ^2^ (1) = 5.7, *p <* 0.05), compared to non-carer control subjects.  The patient mean (range) Beck Depression Inventory (BDI) score was ‘minimal’ according to the BDI criteria, at 6.9 (0 – 24) and it is therefore unlikely that depression was influencing sleep in the majority of individuals.  Unfortunately the BDI was not administered to control subjects in parallel to the sleep questionnaire; therefore the extent to which depression is affecting the scores of control subjects is unknown.  Fewer patients described their sleep as ‘deep’ (χ^2^ (1) = 8.0, *p <* 0.01), ‘long’ (χ^2^ (1) = 8.6, *p <* 0.01), ‘uninterrupted’ (χ^2^ (1) = 5.6, *p <* 0.05) and ‘still’ (χ^2^ (1) = 24.4, *p <* 0.001) and/or as having ‘many dreams’ (χ^2^ (1) =8.8, *p <* 0.01) compared to non-carer control subjects.  There were no significant differences in the reported total hours slept per night, the total number of reported waking times during the night, or reasons for awakening.  

*Motor activity*

      More patients than non-carers reported experiencing a painful muscle cramps in the arms or legs causing them to wake up at night (*p <* 0.01) and repeated jerking or twitching of the arms or legs during sleep (χ^2^ (1) = 10.3, *p <* 0.001), as well as fidgeting a lot in bed (χ^2^ (1) = 27.5, *p <* 0.001).  Furthermore, more patients reported wandering about at night compared to non-carer control subjects (*p <* 0.05), especially in more advanced cases.   

*Abnormal nocturnal behaviour*

      More patients than control subjects reported acting out dreams (*p <* 0.05) and/or injuring themselves or others whilst dreaming compared to the combined control group (*p <* 0.01).  This was not related to disease severity. 

*Other aspects of disturbed sleep*

      More patients reported getting mentally agitated at night (*p <* 0.05) compared to non-carer control subjects and more patients appeared to suffer from nocturia (χ^2^ (1) = 4.9; *p <* 0.05).

A number of aspects of night-time behaviour (numbness, paresthesae or formication of the legs causing sleep problems, or waking up early in the morning with stiff and painful arms or legs) did not differ between HD and control groups, nor were there any differences in sleep apnoea, dreaming, early morning wakening or daytime somnolence and napping.  

In the questionnaire, several questions appeared to show an increased response associated with disease severity using the UHDRS total motor scores of 0-20 compared to those with scores of 41-60, see Table 3.  These questions might in the first instance appear to suggest that they could be sensitive to disease progression, however given the cross-sectional nature of the study, longitudinal conclusions regarding disease progression cannot be made at this point.  In addition to this, it is important to note that the UHDRS motor score is weighted towards the measurement of chorea in patients and this be reflected in questions involving, for example, fidgeting.

Table 3.  Percentage of patients in different disease stages (UHDRS 0-20, 21-40 and >40) who responded as having a disturbance of sleep in the question given.

**Table:** 

	**UHDRS motor score**		
	0-20 (*n* = 24)	21-40 (*n* = 25)	41+ (*n* = 17)
Question	* * *(%) * *number of responses*		
4. On average, how many hours of sleep do you have per night?	16.7	16.0	5.9
5. How long does it usually take you to fall asleep? * One hour or more*	12.5	32.0	29.4
6. How many times do you awaken in a typical nights sleep?	12.5	12.0	11.8
7. Amount of sleep needed changed over 5 years? *I need more *	29.2	36.0	47.1
8. Have difficulty falling asleep at night?	29.2	44.0	35.3
9. Have difficulty staying asleep at night?	29.2	40.0	29.4
10. Wake up for long periods during the night?	25.0	28.0	35.3
11. Wake up early in the morning, unable to return to sleep?	29.2	40.0	41.2
12. Wake up in the night due to full bladder?	41.7	32.0	23.5
14. Repeated jerking/twitching of arms or legs during sleep?	12.5	44.0	52.9
15. Painful muscle cramps in arms or legs that wake you?	25.0	24.0	29.4
18. Get very agitated at night?	29.2	12.0	11.8
24. Fidget a lot in bed?	45.8	72.0	64.7
27. Act out dreams?	16.7	4.0	17.6
29. c) Describe your sleep? *Short *	37.5	28.0	11.8
29. j) Describe your sleep? *Restless*	41.7	52.0	23.5
34. Find you are awake at night, asleep during day?	8.3	16.0	29.4
			

**

**

**

**

**

**

**

**

**

**

**

**

**

**

**

**

**

**

**

**

**

**

**

**

*Scoring system validation*

       The questionnaire was scored to obtain a sleep disturbance score. There was a significant difference between the mean of total sleep disturbance scores in patients and each of the control groups (*p < * 0.001) and between carer and non-carer control groups (*p < * 0.001; Figure 1).  A univariate analysis of variance test revealed a significantly different distribution of individual sleep scores in patients compared to control subjects (f (2,163) = 21.24, *p < * 0.0001).  Further post hoc tests (Dunnett test) showed that there were significant differences between the distribution of patient scores and both control groups (*p < * 0.0001; Figure 2).  This is consistent with the fact that a greater proportion of neurologically normal individuals gained low scores, compared to those of the patients. 

      As a result of these findings, we recommend that the questionnaire be used by clinicians and that out of a total score of 19 points, scores of (0 – 3) represent the ‘normal’ range whilst, scores of (4 – 6) reflect ‘mild’ and scores of 7 and above indicate a ‘significant’ sleep disturbance which may merit further investigation and/or treatment by the clinician. 

Figure 1.  Means of sleep disturbance scores in patients, non-carer controls and carer controls (*** p<0.001).

**Figure:**
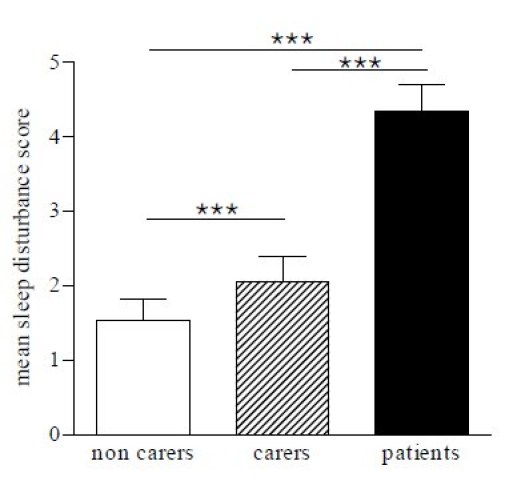

Figure 2. The frequency distribution of sleep disturbance scores ranging from normal, mild and moderate-severe sleep disturbance, in (a) patients, (b) carer  and (c) non-carer control groups.

**Figure:**
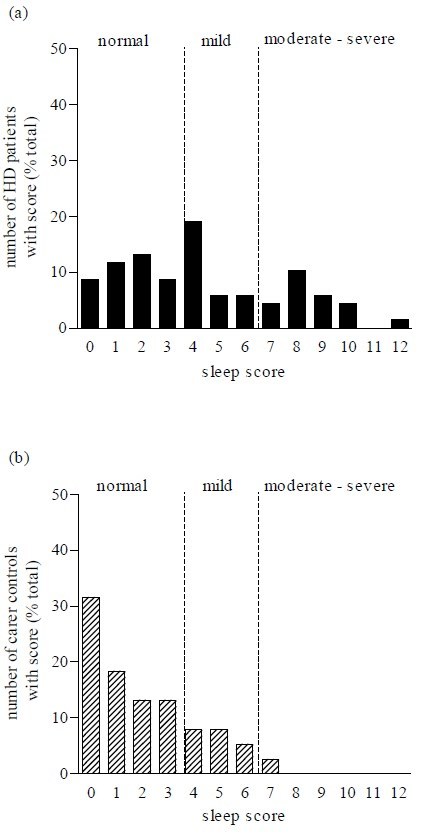

**Figure:**
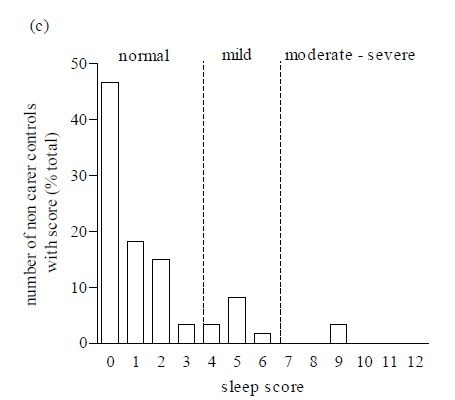

DISCUSSION

       A cross-sectional study of 66 patients with HD using a disease focused questionnaire on sleep revealed several findings.  Firstly, a significantly greater proportion of HD patients reported difficulty in falling asleep and took longer to get to sleep than non-carer control subjects, in agreement with previous findings of increased sleep onset latency in HD[12] [19].  Further analysis did not reveal any significant differences in the reported total hours slept per night, even though historically actual amounts are reported to differ in HD[20] [21]. Secondly more patients reported having difficulty staying asleep at night and fewer patients described their sleep as satisfactory, which agrees with  previous studies reporting a disorder of initiating and maintaining sleep[12], as well as increased wakefulness after sleep onset and decreased sleep efficiency[18] [16] [17] [12].  Thirdly, more patients than non-carers reported experiencing repeated jerking or twitching of the arms or legs during sleep, as well as fidgeting a lot in bed, with more patients reported wandering about at night compared to non-carer control subjects.  These results support previous findings of heightened nocturnal activity in patients with HD compared to control subjects[17].  This could be due to chorea although traditionally CNS-derived movement disorders are said to stop during sleep, although this may only be true for REM sleep. Finally, more patients than control subjects reported acting out dreams and/or injuring themselves or others whilst dreaming compared to the combined control group. suggesting a REM behaviour disorder[22] [23] (as defined by the International Classification of Sleep Disorders (ICSD, 1990)). This disorder has been reported to precede or to co-occur with other neurodegenerative disorders and especially with the α-synucleinopathies such as Parkinson’s disease[24] [25] and Multiple System Atrophy(MSA)[26], but may also be a feature of HD. Indeed, a  recent paper reported a 12% prevalence (three out of 25 HD patients) as having REM behaviour disorder[27].

     A number of aspects of night-time behaviour did not differ between HD and control groups including sensory symptoms in the legs suggestive of RLS, waking up with stiff and painful arms or legs; parasomnias; symptoms of sleep apnoea; upsetting dreaming or daytime somnolence.  

      The current mainstay assessment of patients with HD is the Unified Huntington’s Disease Rating Scale (UHDRS), Independence Score, Total Functional Capacity and Total Functional Assessment, none of which includes questions relating to sleep.  A suitable questionnaire or scale on sleep would be an extremely helpful addition in the assessment process given the abnormalities we found and those reported in previous studies.  For this reason we devised a novel, convenient, simple scoring system utilising a sleep questionnaire which gives a single ‘total points’ score that could be used to give a level of sleep disturbance.  This new questionnaire was validated retrospectively by calculating each respondent’s total HD sleep score and showed a significant difference between the mean total sleep scores in patients, compared to each of the control groups. As a result, we propose that scores of less than three points be considered ‘normal’, whilst scores between four and six reflect ‘mild’ sleep disturbance and scores of seven and above may potentially indicate a ‘significant’ sleep disturbance that merit further investigation and/or treatment.  In our cohort, 35.3% of HD had mild disturbance whilst 26.5% had severe abnormalities, compared to 13.3% and 3.3% respectively, in the normal control population.  Although our scoring system assigned scores to only a subset of the questions, we suggest that the questionnaire should be administered in its entirety for three reasons.  First, it is short; second, the patient cannot predict which questions will be scored, and finally some of the non-scored questions will give the clinician qualitative data about sleep quality on the patient that may be empirically helpful.

    Whilst this study has revealed a number of abnormalities of sleep in HD through the use of a novel questionnaire, there are several limitations to this study.  The first is that our questionnaire has not been validated in other larger groups of patients over time and has only been given to HD patients and ‘control subjects’. It would be informative to see the profile of responses to the questionnaire in other neurodegenerative disorders, such as Parkinson’s disease and Alzheimer’s disease to ascertain whether or not the questionnaire has any disease specificity.  In this respect, further comparisons are needed, including comparisons with control populations to investigate the effects of depression and medication on the sleep questionnaire score and pattern of abnormalities. Finally, our questionnaire is self-reported and HD patients are known to lack insight[28] and thus may not provide an accurate assessment of their situation. However, since HD patients are more likely to underestimate rather than overestimate the extent of their problems, the findings identified by the questionnaire may in fact be more marked than reported.  

    In conclusion, significantly more HD patients were found to experience abnormalities of sleep than control subjects using a novel Sleep Questionnaire which can be easily administered in the clinical assessment of patients. Identification of a disturbance in sleep and circadian rhythm in HD is of great consequence as a disruption of the sleep-wake axis has been found to result in a broad range of interconnected pathologies[29] [30] including poor vigilance and memory[31] [32] [33] reduced mental and physical reaction times[34] [35] reduced motivation, depression[36] [29] and metabolic abnormalities[37] [38].  Disturbed sleep in HD may therefore contribute to the deterioration of the patient’s ability to do activities of daily living and have a significantly deleterious effect on the quality of life of both patients and carers. In this respect, studies to investigate the effects of pharmacological manipulation of sleep disturbances in HD are needed and this questionnaire may provide one convenient tool by which the efficacy of such studies can be assessed.

**Acknowledgments** We wish to thank all of our participating patients and controls for their help and Dr. Gregory Stores for his advice and expertise.

**Funding**

This research was supported by a grant from the CHDI Foundation.**Competing interests**  The authors have declared that no competing interests exist.

- APPENDIX
- Appendix 1 - Sleep Questionnaire
**Figure:**
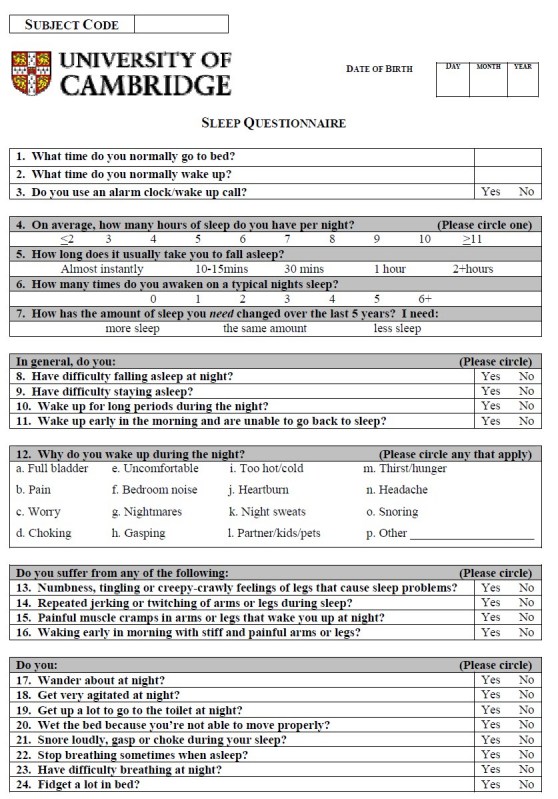

**Figure:**
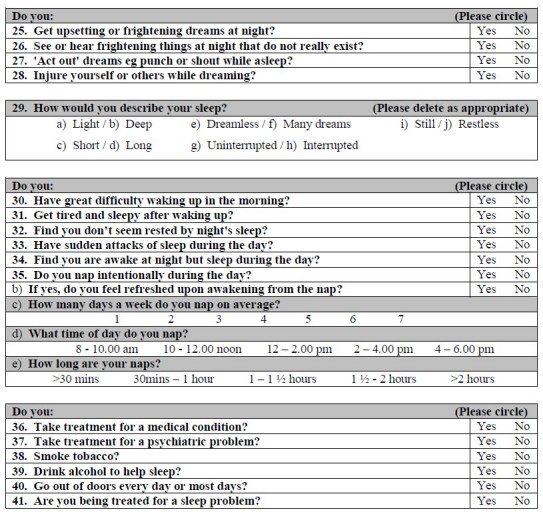
- Appendix 2 - Sleep Questionnaire with suggested scoring
**Figure:**
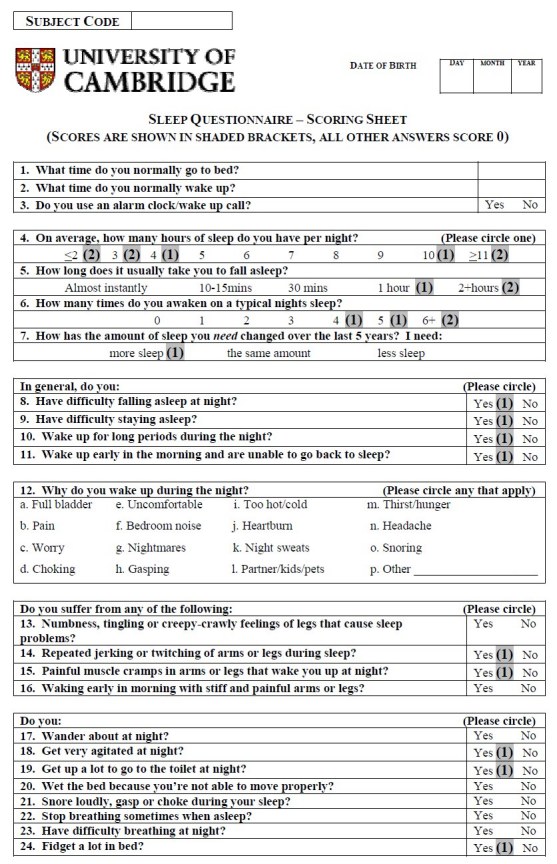
**Figure:**
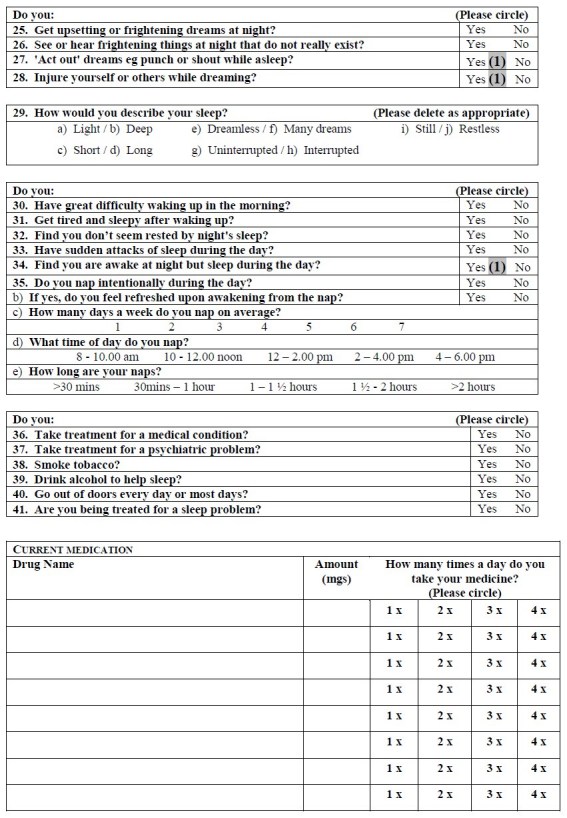

## References

[ref-322109239] Van Someren EJ. Circadian and sleep disturbances in the elderly. Exp.Gerontol. 2000; 35:1229-37. 10.1016/s0531-5565(00)00191-1 11113604

[ref-2816639175] Prinz PN, Vitaliano PP, Vitiello MV et al. Sleep, EEG and mental function changes in senile dementia of the Alzheimer's type. Neurobiol.Aging 1982; 3:361-70. 10.1016/0197-4580(82)90024-0 7170052

[ref-1600911606] Montplaisir J, Petit D, Lorrain D et al. Sleep in Alzheimer's disease: further considerations on the role of brainstem and forebrain cholinergic populations in sleep-wake mechanisms. Sleep 1995; 18:145-48. 10.1093/sleep/18.3.145 7610309

[ref-929571528] Chokroverty S. Sleep and degenerative neurologic disorders. Neurol.Clin. 1996; 14:807-26. 10.1016/s0733-8619(05)70286-3 8923496

[ref-3423957982] Kumar S, Bhatia M, Behari M. Sleep disorders in Parkinson's disease. Mov Disord. 2002; 17:775-81. 10.1002/mds.10167 12210875

[ref-1483623580] Happe S, Berger K. The association of dopamine agonists with daytime sleepiness, sleep problems and quality of life in patients with Parkinson's disease--a prospective study. J.Neurol. 2001; 248:1062-67. 10.1007/s004150170026 12013583

[ref-715185137] Happe S. Excessive daytime sleepiness and sleep disturbances in patients with neurological diseases: epidemiology and management. Drugs 2003; 63:2725-37. 10.2165/00003495-200363240-00003 14664652

[ref-69212180] Vonsattel JP, Myers RH, Stevens TJ et al. Neuropathological classification of Huntington's disease. J.Neuropathol.Exp.Neurol. 1985; 44:559-77. 10.1097/00005072-198511000-00003 2932539

[ref-3229438880] Kremer HP, Roos RA, Dingjan G et al. Atrophy of the hypothalamic lateral tuberal nucleus in Huntington's disease. J Neuropathol.Exp.Neurol. 1990; 49:371-82. 10.1097/00005072-199007000-00002 2141871

[ref-1196567975] Kremer HP, Roos RA, Dingjan GM et al. The hypothalamic lateral tuberal nucleus and the characteristics of neuronal loss in Huntington's disease. Neurosci.Lett. 1991; 132:101-04. 10.1016/0304-3940(91)90443-w 1838577

[ref-1399456708] Taylor N, Bramble D. Sleep disturbance and Huntingdon's disease. Br.J Psychiatry 1997; 171:393. 10.1192/bjp.171.4.393c 9373439

[ref-2263075148] Hansotia P, Wall R, Berendes J. Sleep disturbances and severity of Huntington's disease. Neurology 1985; 35:1672-74. 10.1212/wnl.35.11.1672 2932657

[ref-3405583437] Petit D, Gagnon JF, Fantini ML et al. Sleep and quantitative EEG in neurodegenerative disorders. J.Psychosom.Res. 2004; 56:487-96. 10.1016/j.jpsychores.2004.02.001 15172204

[ref-2102766780] Scott DF, Heathfield KW, Toone B et al. The EEG in Huntington's chorea: a clinical and neuropathological study. J.Neurol.Neurosurg.Psychiatry 1972; 35:97-102. 10.1136/jnnp.35.1.97 PMC493983 4260288

[ref-477668347] Sishta SK, Troupe A, Marszalek KS et al. Huntington's chorea: an electroencephalographic and psychometric study. Electroencephalogr.Clin.Neurophysiol. 1974; 36:387-93. 10.1016/0013-4694(74)90188-6 4140065

[ref-3057807117] Wiegand M, Moller AA, Lauer CJ et al. Nocturnal sleep in Huntington's disease. J.Neurol. 1991; 238:203-08. 10.1007/BF00314781 1832711

[ref-1573408095] Silvestri R, Raffaele M, De Domenico P et al. Sleep features in Tourette's syndrome, neuroacanthocytosis and Huntington's chorea. Neurophysiol.Clin. 1995; 25:66-77. 10.1016/0987-7053(96)81034-3 7603414

[ref-656859450] Mena-Segovia J, Cintra L, Prospero-Garcia O et al. Changes in sleep-waking cycle after striatal excitotoxic lesions. Behav.Brain Res. 2002; 136:475-81. 10.1016/s0166-4328(02)00201-2 12429410

[ref-924260019] Wiegand M, Moller AA, Schreiber W et al. Brain morphology and sleep EEG in patients with Huntington's disease. Eur.Arch.Psychiatry Clin.Neurosci. 1991; 240:148-52. 10.1007/BF02190755 1827599

[ref-2158000020] Hurelbrink CB, Lewis SJ, Barker RA. The use of the Actiwatch-Neurologica((R)) system to objectively assess the involuntary movements and sleep-wake activity in patients with mild-moderate Huntington's disease. J.Neurol. 2005. 10.1007/s00415-005-0709-z 15742112

[ref-1360451065] Morton AJ, Wood NI, Hastings MH et al. Disintegration of the sleep-wake cycle and circadian timing in Huntington's disease. J.Neurosci. 2005; 25:157-63. 10.1523/JNEUROSCI.3842-04.2005 PMC6725210 15634777

[ref-2335371877] Lapierre O, Montplaisir J. Polysomnographic features of REM sleep behavior disorder: development of a scoring method. Neurology 1992; 42:1371-74. 10.1212/wnl.42.7.1371 1620348

[ref-4262421549] Schenck CH, Mahowald MW. REM sleep behavior disorder: clinical, developmental, and neuroscience perspectives 16 years after its formal identification in SLEEP. Sleep 2002; 25:120-38. 10.1093/sleep/25.2.120 11902423

[ref-1711648885] Poryazova RG, Zachariev ZI. REM sleep behavior disorder in patients with Parkinson's disease. Folia Med.(Plovdiv.) 2005; 47:5-10. 16152765

[ref-4133303196] Boeve BF, Silber MH, Ferman TJ. REM sleep behavior disorder in Parkinson's disease and dementia with Lewy bodies. J.Geriatr.Psychiatry Neurol. 2004; 17:146-57. 10.1177/0891988704267465 15312278

[ref-2417781273] Iranzo A, Santamaria J, Rye DB et al. Characteristics of idiopathic REM sleep behavior disorder and that associated with MSA and PD. Neurology 2005; 65:247-52. 10.1212/01.wnl.0000168864.97813.e0 16043794

[ref-728504355] Arnulf, I., Nielsen, J., Lohmann, E., Schieffer, J., Wild, E., Jennum, P., Konofal, E., Walker, M., Oudiette, D., Tabrizi, S., Durr, A., Rapid eye movement sleep disturbances in Huntington disease. Arch.Neurol. 2008; 65: 482-488. 10.1001/archneur.65.4.482 18413470

[ref-4237705310] Ho AK, Robbins AO, Barker RA. Huntington's disease patients have selective problems with insight. Mov Disord. 2006; 21:385-89. 10.1002/mds.20739 16211608

[ref-397695496] Foster RG, Wulff K. The rhythm of rest and excess. Nat.Rev.Neurosci. 2005; 6:407-14. 10.1038/nrn1670 15861183

[ref-4223227834] Carskadon MA. Sleep deprivation: health consequences and societal impact. Med.Clin.North Am. 2004; 88:767-76. 10.1016/j.mcna.2004.03.001 15087215

[ref-2349688897] Born J, Wagner U. Awareness in memory: being explicit about the role of sleep. Trends Cogn Sci. 2004; 8:242-44. 10.1016/j.tics.2004.04.010 15165544

[ref-3695211363] Karni A, Tanne D, Rubenstein BS et al. Dependence on REM sleep of overnight improvement of a perceptual skill. Science 1994; 265:679-82. 10.1126/science.8036518 8036518

[ref-1544206272] Clemens Z, Fabo D, Halasz P. Overnight verbal memory retention correlates with the number of sleep spindles. Neuroscience 2005; 132:529-35. 10.1016/j.neuroscience.2005.01.011 15802203

[ref-3854778905] Jewett ME, Dijk DJ, Kronauer RE et al. Dose-response relationship between sleep duration and human psychomotor vigilance and subjective alertness. Sleep 1999; 22:171-79. 10.1093/sleep/22.2.171 10201061

[ref-3645680608] Tietzel AJ, Lack LC. The recuperative value of brief and ultra-brief naps on alertness and cognitive performance. J.Sleep Res. 2002; 11:213-18. 10.1046/j.1365-2869.2002.00299.x 12220317

[ref-3043578823] Fava M. Daytime sleepiness and insomnia as correlates of depression. J.Clin.Psychiatry 2004; 65 Suppl 16:27-32. 15575802

[ref-1106810239] Spiegel K, Leproult R, L'hermite-Baleriaux M et al. Leptin levels are dependent on sleep duration: relationships with sympathovagal balance, carbohydrate regulation, cortisol, and thyrotropin. J.Clin.Endocrinol.Metab 2004; 89:5762-71. 10.1210/jc.2004-1003 15531540

[ref-1419962940] Scheen AJ, Van Cauter E. The roles of time of day and sleep quality in modulating glucose regulation: clinical implications. Horm.Res. 1998; 49:191-201. 10.1159/000023170 9550124

